# Supplementary material for: Co-evolution of gene transfer agents and their alphaproteobacterial hosts
Source: J Bacteriol. 2024 Jan 19;206(2):e00398-23. doi: 10.1128/jb.00398-23 (PMC10883770; doi:10.1128/jb.00398-23)
Supplement: Supplemental figures — Figures S1 and S2. [file jb.00398-23-s0001.pdf]

Supplementary Figures

for

Co-evolution of gene transfer agents and their alphaproteobacterial hosts

Roman Kogay[1] and Olga Zhaxybayeva[1,2,#]

[1]*Department of Biological Sciences, Dartmouth College, Hanover, New Hampshire, USA*

[2]*Department of Computer Science, Dartmouth College, Hanover, New Hampshire, USA*

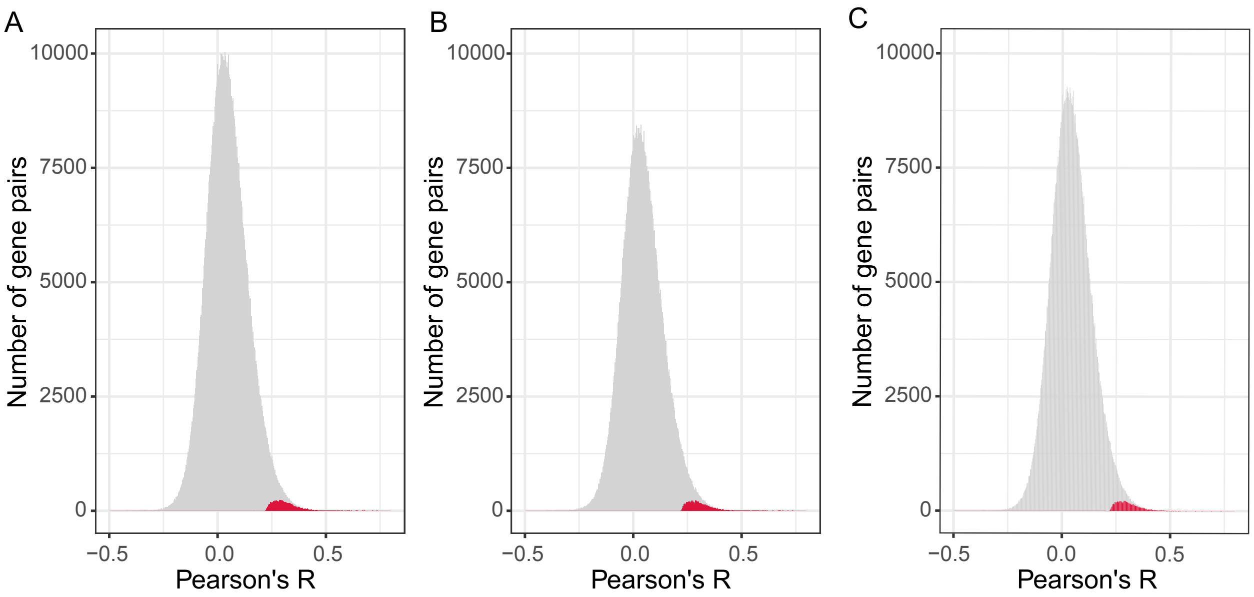

Figure S1. Pearson's correlation coefficients in pairs of genes from (A) *Phaeobacter inhibens*, (B) *Caulobacter crescentus*, and (C) *Dinoroseobacter shibae* genomes. Significantly co-evolving gene pairs (Pearson's R > 0 and p-value <0.05 after Bonferroni correction) are highlighted in red.

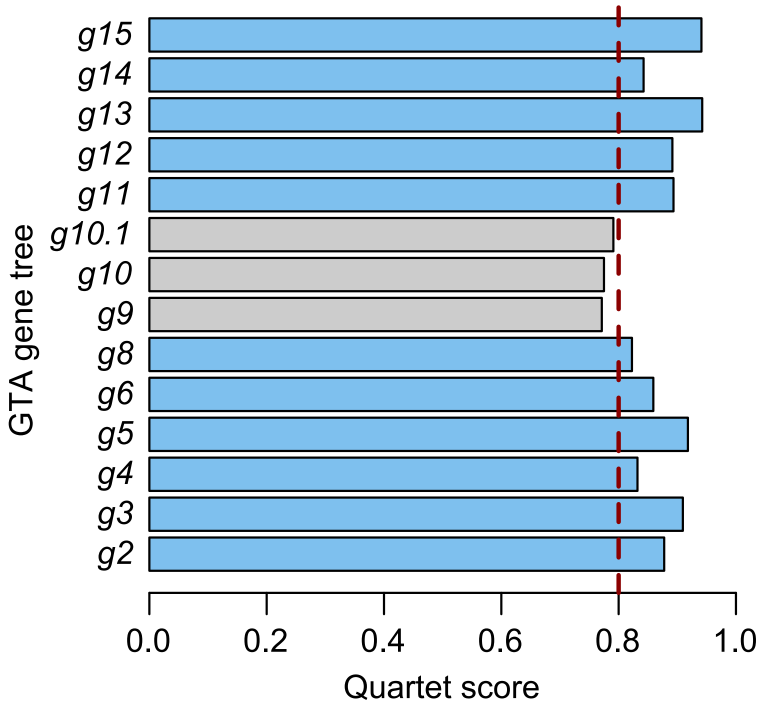

Figure S2. Comparison of GTA head-tail phylogenies and the reference phylogenetic tree. The trees were compared using the quarte score metric (X axis). GTA genes are designated using the gene names of the RcGTA. Dashed red line represent the score cutoff used to define "high congruency". Bars in blue color highlight phylogenies of the reference GTA genes.
